# Management of High Spinal Anesthesia for Cesarean Section in the Semi-Fowler's Position

**DOI:** 10.7759/cureus.93296

**Published:** 2025-09-26

**Authors:** Nathan Martin-Orr, Joseph E Villaluz, Brandon So, Taizoon Q Dhoon, Michael Holland

**Affiliations:** 1 Anesthesiology and Perioperative Medicine, University of California, Irvine (UCI) Health, Orange, USA

**Keywords:** cesarean delivery, dural puncture, failed epidural, high spinal block, neuraxial anesthesia complications, obstetric anesthesia, semi-fowler's position

## Abstract

High spinal anesthesia, a rare but serious complication of neuraxial techniques, occurs when local anesthetic spreads through the cerebrospinal fluid, blocking spinal segments above the intended level. This can lead to significant cardiovascular, respiratory, and neurological effects. These effects include vasodilation, bradycardia, and potential respiratory failure if the phrenic nerve or brainstem is affected. High spinal blocks are particularly concerning in obstetric anesthesia, where they are used for labor analgesia and cesarean deliveries. The ability to quickly and effectively manage this high-risk event can significantly reduce the risk of maternal and fetal morbidity and mortality. In the event of a high spinal, we describe how the use of a semi-Fowler's position can salvage a high spinal and even prevent the need for intubation.

## Introduction

High neuraxial blockade is a rare and serious complication of neuraxial anesthetic techniques. This phenomenon occurs when local anesthetic spreads higher than desired within the cerebrospinal fluid (CSF) or epidural space. Subsequently, this leads to an unintentional block of spinal segments above the intended level. It can cause significant sequelae, including respiratory failure, loss of consciousness, and complete cardiovascular collapse. While spinal and epidural anesthesia are remarkably safe, neuraxial procedures can be complicated by a high spinal or high neuraxial block, as previously stated. Recent studies indicate that the incidence rate of high spinals in obstetric patients is approximately 1 in 4,336 deliveries [1-3].

Identification and management of high spinal blockade should be in the armamentarium of all anesthesia providers. There are specific high-risk scenarios where heightened awareness is prudent, for example, when there is a suspected or known dural puncture with a prior epidural catheter. This specific scenario can be complicated if spinal anesthesia is administered after epidural placement. Furthermore, it is important to consider the intrathecal medication dose, the patient's position following the procedure, and the level of dermatomal spread. In addition to the aforementioned considerations, obstetricians should be present in the operating room in these scenarios. This provides an extra level of preparedness for the possibility of a high spinal block occurrence.

We present the case of a 38-year-old G2P1 female patient who developed a high spinal block following spinal anesthesia placement during an urgent cesarean delivery. This occurred after a previously difficult labor epidural placement and a known dural puncture. The patient was successfully managed, and the cesarean section was performed in a semi-Fowler's position, an approach not commonly discussed in obstetric anesthesia literature. The utilization of semi-Fowler's positioning in managing high spinal blocks during cesarean delivery is currently underexplored but may serve as a valuable management tool.

## Case presentation

The patient was a 38-year-old female, gravida 2 para 1 at 39 weeks and one day gestation, BMI 31.01 kg/m^2^, who presented to the labor and delivery floor for scheduled induction of labor. She had appropriate prenatal care, and her pregnancy was only complicated by gestational diabetes, which was well controlled with insulin.

The anesthesia team was alerted to a labor epidural request. Consistent with our institution's standard practices, the team opted for a labor combined spinal epidural with an Arrow catheter placement. Upon the initial attempt, the Tuohy needle achieved a classical "loss of resistance" with saline, and a spinal dose of 2.5 mg bupivacaine and 5 mcg fentanyl was successfully administered intrathecally after confirmation of CSF return from the spinal needle. After spinal dose administration, the epidural catheter met resistance when attempting to advance despite troubleshooting maneuvers. Subsequent attempts had similar outcomes, regardless of attempting at different spinal levels. The fourth attempt resulted in an accidental dural puncture (ADP) with frank CSF flow. The stylet was replaced, and the Tuohy was removed from the L4-L5 attempt. Another attempt was made at the L3-L4 level. Given the failed attempts to thread the Arrow catheter, a stiffer Braun catheter was successfully threaded on the fifth attempt. Loss of resistance was recorded at 6 cm; the catheter was threaded into the epidural space at 5 cm, and it was secured to the skin at 11 cm. A test dose of 3 cc of 1.5% lidocaine with 1:200,000 epinephrine was administered.

Prior to the test dose, vital signs included a heart rate of 91 bpm, blood pressure of 103/57 mmHg, and an oxygen saturation (SpO_2_%) of 96%. Five minutes after the test dose, the heart rate was 82 bpm, the blood pressure was 121/59 mmHg, and the oxygen saturation (SpO_2_%) was 97%. Given hemodynamic stability, an epidural bolus of 5 cc of 0.125% bupivacaine was administered. The labor epidural infusion was then started at a standard epidural rate of 9 cc/hour of fentanyl 2 mcg per mL/bupivacaine 0.1%. After the epidural bolus, hemodynamics remained stable, with no major fluctuations. Upon immediate re-evaluation, the patient had a partial lower extremity motor blockade with a sensory block to cold at T4. Due to concern for accidental intrathecal catheter despite negative aspiration of CSF, the decision was made to run the epidural pump at a reduced intrathecal rate. The epidural program was run at 2 cc per hour of fentanyl 2 mcg per mL/bupivacaine 0.1%, without bolus function, with plans for reassessment of motor and sensory function.

On follow-up evaluation, the patient developed a receding block height at L2 and inadequate labor analgesia approximately three hours after stopping her initial infusion. As a result, the pump was restarted at the standard epidural rate. Given the complicated epidural course, an anesthetic plan was made. Should conversion to C-section become part of the birthing plan, the labor epidural would be removed, and a spinal would be placed for reliable neuraxial blockade.

Fourteen hours after initial epidural placement, the patient was called for an urgent C-section for NRFHT (non-reassuring fetal heart tones). The anesthetic plan mentioned above was executed. Upon entering the operating room, the patient had a heart rate of 106 bpm, blood pressure of 107/64 mmHg, a mean arterial pressure (MAP) of 78, and an oxygen saturation (SpO_2_%) of 97%. The patient's level was noted at the L2 lateral thigh to be cold temperature despite approximately 107 mL of epidural solution being administered over the course of 14 hours prior to conversion to C-section. Within two hours of presenting in the OR, the patient had not triggered her PCEA (patient-controlled epidural analgesia), and a total of approximately 18 mL of epidural solution had been infused. The epidural was then removed, and the spinal was placed at L3-L4 according to the aforementioned plan. Given the low level of existing anesthesia, the team administered a standard spinal dose (1.6 mL 0.75% bupivacaine, 15 mcg fentanyl, 0.2 mg morphine) in the operating room. Following the administration of the spinal dose, the patient had frequent dermatomal level checks and was quickly positioned supine due to the emergent nature of the cesarean section, with the goal of minimizing the rapid rise of the intrathecal medication.

Shortly after positioning, the patient was hypotensive with a blood pressure of 81/47 mmHg, MAP in the 50s, heart rate of 104 bpm, and oxygen saturation (SpO_2_%) of 90%. The patient then developed shortness of breath, followed by upper extremity weakness and hypophonia, and was placed in left uterine displacement (LUD). She now had a level at C4-C5 with ice testing. Given these symptoms, she was immediately placed in a head-up, semi-Fowler's position, and a nasal cannula was placed for comfort. Saturations prior to nasal cannula were 99%. The OB team was alerted to a high spinal, and upon their evaluation, they felt that they could proceed with the C-section in semi-Fowler's positioning. Vigilant hemodynamic monitoring, hand-grip strength, continued dermatomal testing with ice, and active verbal feedback from the patient were executed every few minutes until delivery to ensure arrest and recession of the spinal blockade. A spinal blockade was placed at 0343, resulting in marked improvement in grip strength, phonation, and subjective improvement in breathing, as reported by the patient at 0405. Hemodynamic stability was maintained with 1.2 L of lactated Ringer's bolus, two separate boluses of IV phenylephrine 100 mcg, and a phenylephrine drip of 10-40 mcg. The estimated quantitative blood loss during the procedure was 895 mL. Uterotonic management consisted of an initial 3 U IV bolus of oxytocin followed by an infusion of 30 U in 500 mL over one hour; no additional uterotonics or antiemetics were required.

The C-section was successfully performed, and by the end of the case, breathing, phonation, and strength had largely improved. The duration of the case from room entry to exit was approximately two hours. The patient remained in semi-Fowler's position until surgical closure. The neonate was delivered in stable condition and did not require resuscitative measures. Upon transferring to the gurney and entering recovery in the PACU (post-anesthetic care unit), the patient remained in a head-up position at a 45-degree angle. The phenylephrine drip was weaned off, and the patient was moving her feet prior to exiting the operating room. The OB team was given strict instructions that the patient should not be laid down flat until she had regained full strength and returned to her baseline motor function. The PACU course was unremarkable, and the patient recovered fully, regaining both strength and respiration without any long-term sequelae. Given her return to pre-neuraxial baseline, no special post-anesthetic management outside of the guidelines at UCI Medical Center was performed.

## Discussion

High spinal anesthesia is a serious complication of central neuraxial techniques, such as spinal and epidural anesthesia. High spinal anesthesia occurs when the local anesthetic spreads to the spinal nerves above the T4 level. This leads to varying degrees of cardiovascular and respiratory compromise depending on the extent of the spread. In cases of total spinal anesthesia, the local anesthetic can reach intracranial levels, resulting in loss of consciousness [4]. Prompt recognition and management of these symptoms is essential to preventing severe outcomes.

Factors contributing to a high spinal blockade include, but are not limited to, the dose and baricity of local anesthetic, patient positioning, intentional/unintentional intrathecal catheter placement, and the presence of a pre-existing epidural block with subsequent spinal dosing [5]. Following the administration of an inadequate epidural, residual epidural volume and pressure may facilitate the cephalad spread of the subsequent intrathecal dose, potentially causing the blockade to reach a higher level than expected [6]. Another recognized cause of high spinal anesthetic includes unknown/known dural puncture during epidural placement [4]. Additional considerations for a high spinal that may also be taken into account include patient height and weight (including large intrauterine pregnancies), the speed of injection, and anatomic factors such as spinal canal morphometry and variations in CSF volume [7,8].

In addition to spinal administration after dural puncture, one of the most well-documented scenarios leading to high spinal blockade is the administration of spinal anesthesia following a failed epidural. The pressure from the prior local anesthetic in the epidural space can pressurize the intrathecal fluid column, causing the additional intrathecal local anesthetic with the SSS (single-shot spinal) to spread cephalad, resulting in a high spinal block. Increased epidural pressure, and consequently CSF pressure, promotes the cephalad spread of intrathecal medications because of reduced CSF volume [9]. The literature indicates a 3% incidence rate of high neuraxial blocks under these conditions [6].

More important than understanding the etiology of a high spinal is the recognition of its presentation, which is paramount to its management. Signs and symptoms of high/total spinal anesthesia can include hypotension and bradycardia due to a blockade of cardiac sympathetic fibers between T1 and T4. Paresthesias, numbness, or weakness in the hands and arms result from a blockade at the C6-C8 levels. Additionally, blockade at these levels may present as shortness of breath due to subsequent weakness of accessory respiratory muscles [4]. Involvement of the spinal nerves between C3 and C5 may present as shoulder weakness, imminent respiratory compromise, hypoventilation, desaturation, and potentially respiratory arrest. As stated above, respiratory involvement is the result of the diaphragmatic innervation of the contributing nerve roots C3-C5. More subtle presentations may include whispered speech due to a lack of ample air excursion needed for phonation. Intracranial spread of local anesthetic to the brain stem can lead to slurred speech, sedation, and loss of consciousness [7,9].

In our case, the patient experienced a high spinal blockade. The patient had a known ADP, which resulted in a reduced intrathecal volume and pressurization of the epidural space due to CSF leakage. This resulted in an unpredictable spread to levels not normally anticipated. Local anesthetic compression of the intrathecal sac is usually the etiology of a high spinal anesthetic following an epidural placement. In this instance, given her presenting dermatomal level of L2, as well as the previously described low-dose PCEA volume and continuous epidural infusion, it is presumed that the local anesthetic is less likely the culprit of her intrathecal sac compression. Unfortunately, despite a previous dermatomal check with confirmation of the level to be at L2, the intrathecal dose resulted in a high spinal presentation.

**Figure 1:**
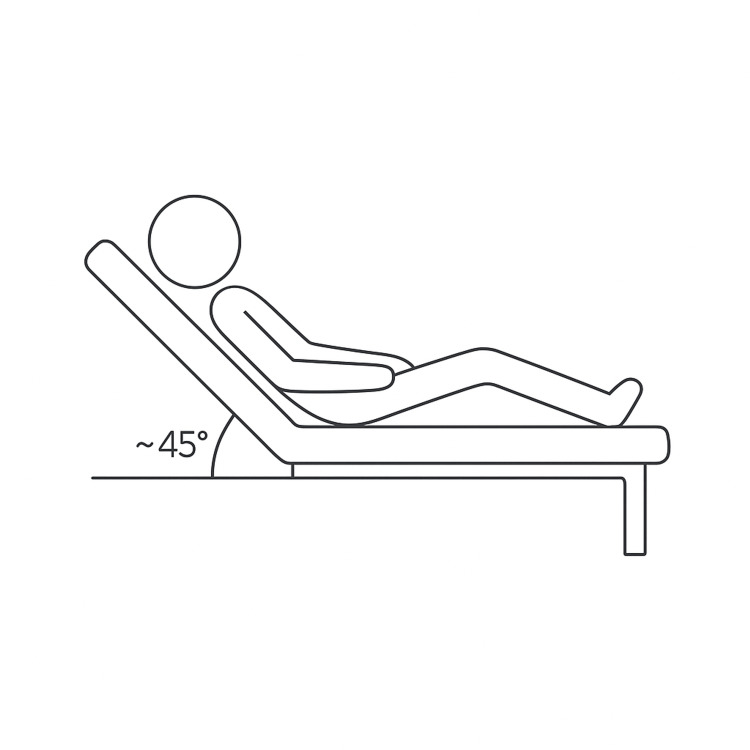
Semi-Fowler's position The image shows a patient positioned in the semi-Fowler's position, lying on their back with the head of the bed elevated to a 45-degree angle. Original artwork created by the authors.

Unique to our case was the intentional implementation of semi-Fowler's positioning (at 45 degrees, as seen in Figure 1) during the C-section, a departure from traditional positioning strategies. Traditional positioning management of high neuraxial blocks during C-section includes lying the patient supine with a 15-degree left lateral tilt [10]. The semi-Fowler's position was selected to manage high spinal anesthesia during the cesarean section by optimizing respiratory function and reducing the risk of aspiration.

This position involves elevating the head and torso at an angle between 30 and 45 degrees, which helps improve lung expansion and ventilation. Enhancing respiratory mechanics can mitigate respiratory compromise that may occur due to the spread of a local anesthetic affecting the phrenic nerve and accessory respiratory muscles. Extreme caution should be taken, as well as vigilant administration of vasopressors, as in our instance, to maintain systemic vascular resistance (SVR) and uteroplacental perfusion. This is important, as placing the patient in a semi-Fowler's position may also lead to a drop in blood pressure initially, prior to the arrest of the rising local anesthetic blockade.

**Figure 2:**
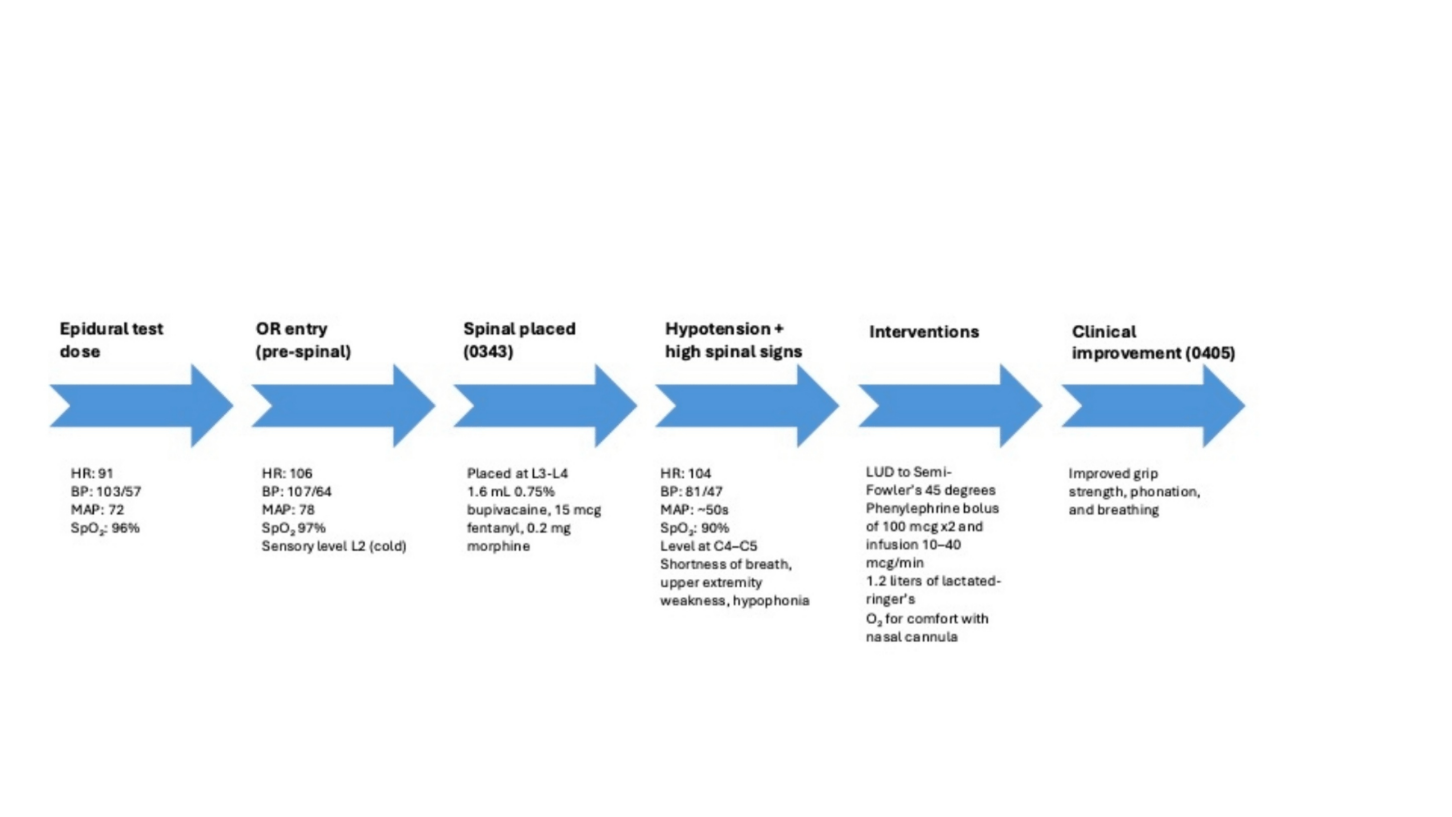
Perioperative timeline with key events and hemodynamics Timeline summarizing major intraoperative milestones (epidural, spinal, hypotension/signs, interventions, improvement) with point vitals to aid correlation with the clinical course. Abbreviations: HR: heart rate; BP: blood pressure; MAP: mean arterial pressure; SpO₂: oxygen saturation; LUD: left uterine displacement.

Additionally, the semi-Fowler's position can help maintain hemodynamic stability by reducing the effects of sympathetic blockade, such as hypotension and bradycardia, and by limiting the cephalad spread of the local anesthetic, thereby leading to its recession. This interpretation is consistent with the rapid regression of the patient's spinal blockade, which improved in approximately 22 minutes of intrathecal dosing (0343-0405). The typical duration of spinal anesthesia with intrathecal bupivacaine lasts between 90 and 150 minutes [11]. Key events and corresponding point vitals are summarized in Figure 2. Semi-Fowler's may also provide hemodynamic advantages over traditional Fowler's positioning due to higher stroke volume and longer ejection time, which suggests enhanced preload preservation and more stable circulatory dynamics [12]. Initially utilized to counteract the high spinal, this positioning was maintained intraoperatively throughout the C-section with coordination from the OB team.

Early recognition of these symptoms was crucial for prompt repositioning into semi-Fowler's and preventing further cephalad spread of local anesthetic. This ultimately facilitated successful cesarean delivery without further complications and avoided emergent intubation. This not only facilitated respiratory mechanics and the recession of the anesthetic level but also maintained cardiac output, as stated above. Additional management measures are supportive, including the use of cardiac vasopressors for hemodynamic stabilization and intubation for respiratory and neurologic compromise.

## Conclusions

Anesthesiologists and obstetric providers must be vigilant in recognizing the signs and symptoms of high spinal anesthesia. Prompt and effective management hinges on close communication during these high-risk events and is crucial to minimizing maternal and fetal morbidity and mortality. Early detection allows for rapid intervention, which is essential in restoring cardiovascular and respiratory stability and preventing further deterioration. The semi-Fowler's position offers promising advantages for both mother and fetus in the management of high spinal anesthesia, highlighting the need for further investigation in obstetric anesthesia practice. We acknowledge this is a preliminary observation and not a definitive management recommendation. Although the semi-Fowler's position poses counterintuitive practices in obstetric anesthesia, such as a decrease in SVR and uteroplacental perfusion post-neuraxial blockade, this may be a useful salvage technique in a high spinal event. This does not negate the need for conventional management of high spinal blockade, including fluid boluses, vasopressor administration, and the potential for intubation in the event of respiratory arrest. Maintenance of maternal and fetal hemodynamics is paramount to all management strategies, regardless of patient positioning.

## References

[1] D'Angelo R, Smiley RM, Riley ET, Segal S (2014). Serious complications related to obstetric anesthesia: the serious complication repository project of the Society for Obstetric Anesthesia and Perinatology. Anesthesiology.

[2] Toledano RD, Leffert L (2021). What's new in neuraxial labor analgesia. Curr Anesthesiol Rep.

[3] Beenakkers IC, Schaap TP, van den Bosch OF (2024). High neuraxial block in obstetrics: a 2.5-year nationwide surveillance approach in the Netherlands. Anesth Analg.

[4] Sivanandan S, Surendran A (2019). Management of total spinal block in obstetrics. Obstet Anaesth.

[5] Yentis SM, Lucas DN, Brigante L (2020). Safety guideline: neurological monitoring associated with obstetric neuraxial block 2020: a joint guideline by the Association of Anaesthetists and the Obstetric Anaesthetists' Association. Anaesthesia.

[6] Einhorn LM, Habib AS (2016). Evaluation of failed and high blocks associated with spinal anesthesia for cesarean delivery following inadequate labour epidural: a retrospective cohort study. Can J Anaesth.

[7] Ituk U, Wong C (2025). Overview of neuraxial anesthesia. UpToDate.

[8] Doelakeh ES, Chandak A (2023). Risk factors in administering spinal anesthesia: a comprehensive review. Cureus.

[9] Beck GN, Griffiths AG (1992). Failed extradural anaesthesia for caesarean section. Complication of subsequent spinal block. Anaesthesia.

[10] Befkadu A, Timerga S, Mihretu F, Seyoum F, Alimawu A (2024). The sitting versus lateral position during induction of spinal anesthesia for elective cesarean delivery on block characteristics and severity of hypotension: a prospective randomized clinical trial. Int J Surg Open.

[11] Olawin AM, Das JM (2025). Spinal anesthesia. StatPearls [Internet].

[12] Kubota S, Endo Y, Kubota M, Ishizuka Y, Furudate T (2015). Effects of trunk posture in Fowler's position on hemodynamics. Auton Neurosci.

